# Ototoxicity of Topical Antibiotic Ear Drops in Chronic Suppurative Otitis Media in Humans: A Review of the Literature

**DOI:** 10.7759/cureus.32780

**Published:** 2022-12-21

**Authors:** Syed Zohaib Maroof Hussain, Syed S Hashmi, Asad Qayyum

**Affiliations:** 1 Ear, Nose, and Throat (ENT), Peterborough City Hospital, Peterborough, GBR; 2 Otolaryngology, Peterborough City Hospital, Peterborough, GBR; 3 Otolaryngology - Head and Neck Surgery, Peterborough City Hospital, Peterborough, GBR

**Keywords:** sensorineural hearing loss, cochleotoxicity, vestibulotoxicity, ototoxicity, ototopical preparations, antibiotic ear drops

## Abstract

An electronic search of the literature was performed for reported cases of ototoxicity associated with the use of topical antibiotic ear drops in humans. The dosage, duration, and type of ototoxic preparations involved were recorded. Due to the scant quantity of low-quality information that is currently available, there was uncertainty about the usefulness of topical antibiotics in enhancing the resolution of ear discharge in patients with chronic suppurative otitis media. However, despite this uncertainty, there are some data to show that using topical antibiotics in comparison to a placebo or in conjunction with a systemic antibiotic may be useful. Additionally, there is ambiguity regarding the relative efficacy of various kinds of antibiotics; it is impossible to say with absolute certainty whether quinolones are superior to or inferior to aminoglycosides. Although the adverse impact profiles of these two classes of chemicals varied, there are not enough data from the included trials to draw any conclusions about them. Negative effects were generally underreported.

## Introduction and background

Chronic suppurative otitis media (CSOM) is a long-standing infection of part or whole of the middle ear cleft characterized by chronic/recurrent otorrhoea and permanent perforation. The traditional management of this condition has included an aural toilet, antibiotic ear drops, systemic antibiotics, water precautions, and/or surgery. Various treatments like some forms of aural toileting and antiseptic washouts can be administered by primary healthcare providers or by the patients themselves, but in most nations, antibiotic medication requires a doctor's prescription [1]. There is a range of practices regarding the kind of surgical intervention that should be taken into consideration and the timing of the intervention [1]. Surgical techniques to repair the tympanic membrane are an option in situations where complications occur or in patients who have failed to respond to other treatments [1]. Additionally, surgical interventions may not be accessible or readily available in all settings [1]. Since systemic antibiotics may cause acute side effects, including gastrointestinal discomfort, topical antibiotics are frequently chosen over oral antibiotics [1]. The link between the misuse of systemic antibiotics and rising bacterial resistance among infections acquired in hospitals and the population raises wider concerns [2-4].

It is generally accepted that in the presence of an intact tympanic membrane, topical ototoxic antibiotic ear drops are safe to use. However, inner ear damage is possible if the drug enters the middle ear cavity via a perforation. Indeed, increasing numbers of otorhinolaryngologists use intratympanic gentamicin to ablate vestibular function [5]. The advantage of topical administration over systemic application is that the former may give a high concentration of antibiotic to the afflicted area while the latter is absorbed and dispersed throughout the body [1]. However, if the tympanic membrane hole is tiny or there is a lot of uncleanable mucopurulent discharge in the ear canal, the penetration of topical antibiotics into the middle ear may be hindered [1]. The achievement of compliance with topical dosage in both children and adults may also be challenging. Systemic antibiotics may be preferable in some circumstances [1].

To combat the most frequently grown microorganisms in chronic otitis media (Staphylococcus aureus and Pseudomonas aeruginosa), topical broad-spectrum antibiotics are usually utilized. Examples include second-generation quinolones and aminoglycosides [6]. Antibiotics for CSOM that specifically target Pseudomonas aeruginosa might be superior to those that do not [1]. However, if administered within the therapeutic range, dose and treatment duration are less likely to have an impact on relative effectiveness [1]. In most cases, treatment must last at least five days, and uncomplicated infections can usually be resolved in one to two weeks [1]. To check for recurrence of discharge, a longer follow-up may be required in some patients (more than four weeks), as it sometimes takes more than two weeks for the ear to become dry [1]. When using topical antibiotics or their excipients, the outer ear's skin may get irritated chemically or allergically, causing local discomfort, ear pain, or itching. This can also happen because of the topical antibiotics' or their excipients' use. As a first-line therapy for CSOM, several experts advise using topical antibiotics (without steroids). Differing professional organizations and Ear, Nose, and Throat (ENT) experts from various nations hold different perspectives on the safety of topical antibiotics for the treatment of CSOM, particularly the use of aminoglycosides topically. Even with the use of topical aminoglycosides, there are still worries related to the local toxicity to the inner ear (ototoxicity) [7,8]. Studies in both adult and pediatric populations have shown no ototoxicity with fluoroquinolones (namely, ciprofloxacin and ofloxacin [9]. It is important to recognize that most of the published reports have focussed solely on cochleotoxicity and ignored possible vestibulotoxicity. There remains considerable controversy and confusion regarding the existence and significance of topical ototoxicity, the role of concomitant middle ear disease, and the relative safety of prescribing topical aminoglycoside drops in patients with tympanic membrane perforations. Two major conclusions drawn by Tange RA et al. in 2001 from the Swiss Questionnaire and American studies were: 80% of ENT surgeons questioned felt the risk of hearing loss caused by otitis media was greater than the risk of ototoxicity by aminoglycoside ear drops and 75% of otolaryngologists considered the usage of aminoglycoside ear drops safe in recently operated ears [10].

A computerized search was performed. All the articles available in English on human ototoxicity between 1989 and 2022 on Medline, PubMed, and Google Scholar were included. One Cochrane review was found. The PICO (Patient, Intervention, Comparison, and Outcome) strategy was used to find the appropriate articles (Table 1). The keywords used for the search were: topical ear drops, topical antibiotic ear drops, ototopic preparations, ototoxicity, vestibulotoxicity, cochleotoxicity, and sensorineural hearing loss (Tables 2, 3).

**Table 1:** PICO strategy for searching articles PICO: Patient, Intervention, Comparison, and Outcome

PICO strategy for searching articles	
Population	Patients with chronic suppurative otitis media
Intervention	Topical antibiotic eardrops
Comparison	Placebo OR systemic antibiotics OR oral antibiotics
Outcome	Ototoxicity OR cochleotoxicity, sensorineural hearing loss

**Table 2:** Ototopical preparations

Anti-infective preparations	Anti-inflammatory preparations
Chloromycetin	Betamethasone
Clioquinol	Dexamethasone
Clotrimazole	Flumetasone
Colistin sulfate	Hydrocortisone
Acetic acid	Prednisolone
Framycetin sulfate	Triamcinolone acetate
Polymixin B	
Gentamicin	
Neomycin sulfate	
Sulfacetamide	
Tobramycin	
Ciprofloxacin	

**Table 3:** Types of ototoxicity

Types of ototoxicity	
Cochlear Toxicity	Vestibular Toxicity
Tinnitus - decreased sensorineural hearing thresholds initially affecting the high frequencies before involving others	Ataxia, Oscillopsia, Imbalance, Vertigo

## Review

Methods to assess vestibulocochlear damage

Different authors have used different methods to measure levels of cochlear and vestibular impairments. Cochlear toxicity has been measured using pure tone audiometry and speech thresholds and electrocochleography. In contrast, there are a few reports of more complex methods of measuring vestibular toxicity. The tests included are head shake maneuver, electronystagmography with caloric stimulation, tandem gait test, Romberg’s test, and posturography. Rotational chair tests and Snellen’s charts (used for testing oscillopsia) have been used in severe bilateral cases.

Review of literature

Hui Y et al. (1997) reported two cases of high-frequency sensorineural hearing loss attributed to gentamicin, framycetin sulfate, and cortisporin (neomycin and polymyxin B sulfates, and hydrocortisone otic solution) ear drops, used for several years. These patients had tympanostomy tubes or perforations with discharge. No comment was made about vestibular symptoms [11].

Linder TE et al. (1995) carried out a retrospective review of 134 patient charts treated with topical antibiotic ear drops between 1953 and 1995 for chronic suppurative otitis media. Two cases were reported with profound sensorineural hearing loss “directly attributable to topical ear drops (framycetin and polymixin containing ear drops)”. Ten other suspected cases were insufficiently documented. This study showed the incidence to be somewhere between 2% and 10%. No comment was made on vestibulotoxicity [12].

Podoshin L et al. (1989) compared patients treated with an otic mixture containing neomycin, polymyxin B, and dexamethasone with a control group of 26 getting only dexamethasone. They were followed up for one to two years. They divided the patients into three treatment groups: 33 with long remissions, 69 with short remissions, and 22 treated for one year continuously. Those treated continuously had a hearing loss of 10 dB compared to 3.6 dB in patients treated with short remissions and 1.8 dB with long remissions. Control patients improved by -0.9 db. This is the only controlled trial on humans in the literature in the past years. It shows a slight risk of hearing damage in patients whose symptoms require long-term/continuous treatment. No vestibular assessments were discussed [13].

Force RW et al. (1995) carried out a trial to assess the safety and efficacy of ototopical ciprofloxacin hydrochloride (Ciloxan). Ciprofloxacin was not detected in the plasma level of any child and no adverse effects were noted [14]. Esposito S et al. compared the treatment of chronic otitis media with topical and oral Ciprofloxacin. There were no side effects noted. The investigators concluded that locally administered ciprofloxacin is more effective in curing chronic otitis media than oral ciprofloxacin alone [15].

Ozagar A et al. (1997) performed a prospective randomized trial using ciprofloxacin and gentamicin ear drops in 40 patients (20 in each group) with chronic otitis media. The medicines were used for 10 days. No signs of cochlear or vestibular toxicity were noted. All patients using ciprofloxacin showed an improvement while only six of the 20 using gentamicin showed any clinical response [16].

Wong DL et al. (1997) reported signs of cochleotoxicity in five patients (three ears) who were prescribed gentamicin ear drops for a period varying from 10 days to two months. These patients had tympanic membrane perforations. All the patients had sensorineural hearing loss, of which three were mild to moderate and two were moderate to severe. Case 1 developed vertigo three weeks after the treatment. Case 2 developed a unilateral vestibular disturbance. The other three patients developed signs of vestibulotoxicity following the use of the drug for weeks to months [17].

Marais J et al. (1998) reported nine patients (12 ears in total) who were treated with Gentamicin ear drops for a period of five days to 14 weeks for chronic otitis media with and without suppuration. Vestibulotoxicity was noted in six patients unilaterally and three patients bilaterally. In this study, four patients also developed high-frequency sensorineural hearing loss [18]. 

Bath AP et al. (1999) documented 16 cases (22 ears in total) of ototoxicity in patients with perforations of the tympanic membrane. All of these patients had developed vestibulotoxicity, possibly related to topical drops containing gentamicin/steroid-containing ear drops. The ear drops were used for an average of 31.5 days (range 7-112 days). An additional case was presented reporting intentional ablation of vestibular function using commercially available gentamicin ear drops. No cochleotoxicity was reported [19].

The above-mentioned studies have all been reported from Toronto and it is not clear if the report of Bath is an extension of the series published by Marais J (1998), et al.

In 2001, the Canadian Adverse Drug Reaction Monitoring Programme reported 18 domestic suspected reports of ototoxicity (including published cases) associated with the use of Garasone® ear drops (gentamicin) in the presence of tympanic membrane perforation or the presence of tympanostomy tubes. Of these, 16 were of vestibular toxicity and only two involved hearing loss. In most cases, the condition being treated was chronic otorrhoea. The duration of treatment ranged from five days to a few months. In six patients, the treatment was less than one week [20].

Rutka J et al. (2002), in a review, concluded that the toxic effect of locally used gentamicin is actually delayed and usually occurs a few days after the treatment is stopped. The antibiotic has to be absorbed through the round window membrane, travel from the basal turn of the cochlea, and through all its turns into the otolith organs and the semicircular canals where it does the damage. He stated that there was now incontrovertible evidence of 29 patients referred to him with topical gentamicin ototoxicity, using highly sensitive methods of vestibular toxicity detection, including careful history, proper otoneurological examination, and analyzing laboratory data. His patients had used commercially available gentamicin preparations for an average of 16 days before they developed signs of toxicity in the presence of tympanic membrane perforation. Out of the 29 patients, nine were bad enough to have persisting oscillopsia and or ataxia, seven of the nine never returned to work and five were eventually confined to wheelchairs. He did not however comment if the more severe patients had longer courses or whether these patients were merely more sensitive to the effects of ototopical preparations [21].

Rutka J et al. (2002) also found using pre- and post-treatment electronystagmography and audiogram results that patients with Meniere’s disease were given instructions to instill five to six drops three times a day in the affected ear until they became vertiginous for two days continuously, had almost identical findings to those who had a shorter higher concentration of gentamicin given. Well over 50% of these patients had no response to ice-water caloric (i.e., a profound suppression of vestibular function) [21].

Nawasreh O et al. (2001) examined the effectiveness of ciprofloxacin hydrochloride and gentamicin sulfate in treating chronic otitis media. Two groups of 88 patients with chronic suppurative media, ranging in age from nine to 62, were assigned at random. In the first group, ciprofloxacin hydrochloride was applied topically to 48 patients while local gentamicin sulfate was applied topically to 40 patients in the second group. Of the 48 patients who received ciprofloxacin hydrochloride, 42 were cured while the treatment failed in six of the patients. While 28 patients in the gentamicin group did not exhibit any clinical or bacteriological improvement, 12 of the patients were cured. Treatment of an acute exacerbation of CSOM with topical ciprofloxacin is safer, more effective, and more efficient than treatment with topical gentamicin [22].

Kaygusuz I et al. (2002) found that to treat CSOM, ciprofloxacin, and tobramycin had comparable degrees of effectiveness. The duration of treatment was shortened by adding dexamethasone to ciprofloxacin [23].

Ramos A et al. (2003) found Pseudomonas aeruginosa and Staphylococcus aureus were the most frequently isolated microorganisms [24]. Nineteen ciprofloxacin-resistant strains were discovered. The topical administration groups showed a superior therapeutic response. When ciprofloxacin and fluocinolone were applied topically, a difference was only seen in the groups of individuals with cholesteatoma and persistent middle ear infections with bone reabsorption. Concluding that topical forms of ciprofloxacin treatment in active infection with chronic middle ear disease produce better outcomes than oral dosing. In situations of persistent middle ear infection and cholesteatoma infection, the use of topical fluocinolone improves the outcomes [24].

Liu J (2003) et al. found the rifampicin solution to be effective in CSOM [25]. Otorrhea vanished, as well as mucosal hyperemia of the tympanic membrane and tympanic cavity. No otorrhea complaints were made, there was no obvious purulence in the ear canal or tympanic cavity, and the tympanic membrane and canal only had non-visible or mild hyperemia. When it comes to clearing up ear discharge, topical ofloxacin is preferable to topical gentamycin in the management of CSOM [25].

Asmatullah et al. (2014) followed 134 patients following their discharge for more than three months in a randomized controlled experiment and were randomly assigned to two groups, each of which had 67 patients [26]. Patients in group A received 0.3% of ofloxacin four drops three times daily for 10 days while patients in group B received 0.3% of gentamycin four drops three times daily. Patients were observed for two weeks following therapy for otoscopy, evaluation under a microscope, and assessment of ear complaints [26].

Siddique W et al. (2016) studied, which comprised 186 patients with a diagnosis of CSOM. Patients were assigned at random to either group I, which received topical eardrops of ciprofloxacin (n = 93), or group II, which received topical eardrops of Neomycin (n = 93). At the follow-up examination, the absence of discharge and congestion served as an indicator of success. When CSOM was treated, topical ciprofloxacin performed better than topical Neomycin [27].

Although there is abundant data available examining the toxicity of ototoxic antibiotic ear drops in animal models, there is relatively little objective information regarding eardrop toxicity in humans. Much of the current literature addressing topical therapy for chronic middle ear disease consists of case reports that are of limited value.

Much of the current literature addressing topical therapy for chronic middle ear disease consists of non-RCTs, which limits their reliability. The value of animal studies can be questioned, as there are marked anatomical differences between animals and humans that may account for the discrepancies in their susceptibilities to ototoxicity [28].

The amount of medication reaching the middle ear is also dependent on the site and size of the tympanic perforation and the amount of active ear discharge [19]. Poor Eustachian tube function may not allow the eardrops to enter the middle ear cleft or conversely a patent tube may allow the drug to drain away [19]. The presence of pus or fluid within the middle ear may physically block or dilute the concentration of the eardrops [19]. Increased vascularity of the middle ear mucosa, as occurs in chronically diseased ears may encourage the uptake of drops into the general circulation or the inner ear [19].

Similarly, a study published in 2018 concluded that it is important to adopt robust ototoxic monitoring in the case of ototoxic medications. In addition, a clinical trial in this area is of significant importance, and it can help develop international guidelines for ototoxicity monitoring [29]. 

In a review of the literature, ototopical antibiotic drops confined to humans and the English language, only 34 found a total of 54 cases of gentamicin vestibular toxicity and of these patients, 24 also developed cochlear toxicity. Similarly, with neomycin-based preparations, they found 11 cases of cochlear and 2 cases of vestibular toxicity. Their strict exclusion criteria meant that a significant number of reports on the subject very relevant to the subject in our view were not included [30]. 

In view of the published evidence of clinical ototoxicity (especially from Canada), we feel the time has come for clinicians treating patients with chronic suppurative otitis media to use aminoglycoside drops with caution and a clinical review of their potential complications.

If in doubt, the use of appropriate quinolones (eg ofloxacin, ciprofloxacin) should be considered. Harris AS (2016), et al. reported that quinolones carry a lower risk of ototoxicity compared to aminoglycosides. Furthermore, they are equal or more effective in treating CSOM. They also recommended the prophylactic use of quinolones in post-myringotomy. Hence, topical quinolones should be considered a first-line treatment for these patients [31].

The advice of the British National Formulary has also changed over the years. Up to 1988, its advice was that ear drops should not be prescribed in the presence of ear drum perforation. In 1991, there was a slight modification in the protocol: in the presence of a perforation, many specialists may use ototopic drops but with caution. In 1998, the protocol was changed again based on reports of ototoxicity. The Committee on Safety of Medicine (CSM) issued a reminder that treatment with ear drops in the presence of tympanic membrane perforations is contraindicated.

The medicine with proven toxicity in humans is aminoglycosides because of its role in chemical labyrinthectomy. Therefore, this ototoxic drug in particular should be used with extreme caution. The majority of the studies performed have focused on gentamicin and not much has been stated about the toxicity of Soframycin, though sporadic reports are present in the literature suggesting a potential for ototoxicity.

Similarly, Phillips JS et al. (2007) conducted a review, in which they concluded that aminoglycosides should be given only in presence of an obvious infection with no longer than two weeks in the case of a perforated ear drum. In addition, where possible, pre-treatment audiometry should be performed as well. The Council of ENT-UK fully endorsed those recommendations and was accessible via the ENT-UK website [7].

There are ototopically safe drugs, such as ciprofloxacin and ofloxacin, which were not licensed for aural use in the UK but were available for the same in the US. In the UK, they were available as topical ophthalmic solutions. There were no clear guidelines or recommendations for quinolone use in the UK as mentioned by Phillips JS et al. as well [7].

Furthermore, currently, the British National Formulary (BNF) has updated the guidelines and most recent data suggest the use of ciprofloxacin ear drops in children above one year of age. The following topical ear preparations are available in the UK (Table 4).

**Table 4:** Topical ear preparations available in the UK for treating otitis externa

Group	Products
Astringent/acidic preparations	
Astringent/Acidic	Aluminum acetate 8%* and 13% drops; combined acetic acid 8.25% with aluminum acetate and aluminum acetotartrate 1.8% spray (Otinova®)† Acetic acid 2% spray (Earcalm®)†
Corticosteroid preparations	
Corticosteroid: lower potency	Prednisolone sodium phosphate 0.5% drops (Predsol®)
Corticosteroid: higher potency	Betamethasone sodium phosphate 0.1% drops (Betnesol®, Vista-methasone®)
Antibiotic preparations	
Aminoglycoside	Gentamicin 0.3% drops (Genticin®)
Fluoroquinolone	Ciprofloxacin 2mg/ml (Ciloxan®, Cetraxal®)
Antifungal preparations	
Antifungal	Clotrimazole 1% solution (Canesten®)
Combined corticosteroid and antibiotic preparations	
Ciprofloxacin	Ciprofloxacin 0.3%, dexamethasone 0.1% ear drops Fluocinolone 0.25mg/ml, ciprofloxacin 3mg/ml (Cetraxal plus®)
Aminoglycoside with corticosteroid	
Neomycin	Betamethasone sodium phosphate 0.1%, neomycin sulfate 0.5% (drops: Betnesol-N®, Vista-Methasone N®) Hydrocortisone 1%, neomycin sulfate 3400 units, polymyxin B sulfate 10,000 units/mL (drops: Otosporin®) Prednisolone sodium phosphate 0.5%, neomycin sulfate 0.5% (drops: Predsol-N®) Dexamethasone 0.1%, neomycin sulfate 3250 units/mL, glacial acetic acid 2% (spray: Otomize®)
Gentamicin	Hydrocortisone acetate 1%, gentamicin 0.3% (drops: Gentisone HC®)
Framycetin	Dexamethasone 0.05%, framycetin sulphate 0.5%, gramicidin 0.005% (drops: Sofradex®)
Combined corticosteroid and antibiotic/antifungal preparations	
Clioquinol with corticosteroid	Flumetasone pivalate 0.02%, clioquinol 1%
*Aluminum acetate 8% can be made by diluting 8 parts aluminum acetate ear drops (13%) with 5 parts purified water (must be freshly prepared before use). †Available to buy over-the-counter.	

But this has some limitations as per the committee [32,33]:

- Topical aminoglycoside preparations are contraindicated in people with a perforated tympanic membrane due to the risk of ototoxicity but may be used on the advice of a specialist.

- Prescribe a non-ototoxic preparation if the person has a known or suspected perforation of the tympanic membrane, including a tympanostomy tube in situ.

- If there is a history of suspected contact sensitivity to a topical ear preparation, advise avoiding all preparations with the same class of drug associated with the reaction. For example, if neomycin is thought to have caused a sensitivity reaction, all preparations containing aminoglycosides should be avoided.

Ototoxicity from intravenous aminoglycoside administration is well-documented. We don’t find in-depth and clear answers for aminoglycoside-containing topical preparations. Our review suggests that aminoglycosides containing ear drops (such as gentamicin or neomycin) can occasionally cause hearing loss if administered in a perforated dear drum for a long period of time. There is also evidence for vestibulotoxicity from gentamicin topical drops.

However, still many things are unclear about ototopicals and their toxicity such as hearing toxicity caused by gentamicin when given topically, but rarely when given systemically, how can one monitor for topical antibiotic ototoxicity?

## Conclusions

The findings of this review indicate that there is very low certainty evidence that, the use of topical antibiotic therapy may improve the short-term clearance of ear discharge in patients with CSOM. The effects of topical antibiotics (without steroids) for high-risk groups like immunocompromised individuals or indigenous populations are not well-understood due to a lack of sufficient evidence. However, their potential ototoxic effects are still a matter of controversy. Ciprofloxacin and ofloxacin have now been recognized as safe for the cochlea and they are effective against Pseudomonas, making them a suitable choice in CSOM.

However, the vestibulotoxic effects of most of the topical antibiotics were found to be underreported. These questions currently cannot be answered with any degree of assurance due to the low level of certainty in the evidence that is available. There is unquestionably room for additional studies that evaluate the effectiveness of topical antibiotics for individuals with CSOM, as well as studies that evaluate the class of antibiotic and the dosage/duration. Although topical quinolones and topical aminoglycosides have been examined in most studies, the certainty of the evidence is still very poor (GRADE (Grading of Recommendations, Assessment, Development, and Evaluations)) for this comparison. Effectiveness and negative consequences over the long run are also significant. Health services should create prospective databases for patients with CSOM in addition to therapeutic trials so that they can record the long-term outcomes for the resolution of discharge, adverse effects, and hearing outcomes for those getting therapy.
